# American Telemedicine Association’s Principles for Delivering Telerehabilitation Services

**DOI:** 10.5195/ijt.2017.6232

**Published:** 2017-11-20

**Authors:** TAMMY RICHMOND, CHRISTOPHER PETERSON, JANA CASON, MIKE BILLINGS, EVELYN ABRAHANTE TERRELL, ALAN CHONG W. LEE, MICHAEL TOWEY, BAMBANG PARMANTO, ANDI SAPTONO, ELLEN R. COHN, DAVID BRENNAN

**Affiliations:** 1 GO2CARE, LOS ANGELES, CA; 2 HARTFORD HEALTHCARE, HARTFORD, CT; 3 AUERBACH SCHOOL OF OCCUPATIONAL THERAPY, SPALDING UNIVERSITY, LOUISVILLE, KY; 4 INFINITY REHABILITATION, WILSONVILLE, OR; 5 NICKLAUS CHILDREN’S HOSPITAL, MIAMI CHILDREN’S HEALTH SYSTEM, MIAMI, FL; 6 DOCTOR OF PHYSICAL THERAPY PROGRAM, MOUNT ST. MARY’S COLLEGE, LOS ANGELES, CA; 7 VOICE & SWALLOWING CENTER OF MAINE, WALDO COUNTY GENERAL HOSPITAL, BELFAST, ME; 8 DEPARTMENT OF HEALTH INFORMATION MANAGEMENT, SCHOOL OF HEALTH AND REHABILITATION SCIENCES, UNIVERSITY OF PITTSBURGH, PITTSBURGH, PA; 9 DEPARTMENT OF HEALTH INFORMATION MANAGEMENT, SCHOOL OF HEALTH AND REHABILITATION SCIENCES, UNIVERSITY OF PITTSBURGH, PITTSBURGH, PA; 10 DEPARTMENT OF COMMUNICATION SCIENCE AND DISORDERS, SCHOOL OF HEALTH AND REHABILITATION SCIENCES, UNIVERSITY OF PITTSBURGH, PITTSBURGH, PA; 11 TELEHEALTH INITIATIVES, MEDSTAR INSTITUTE FOR INNOVATION, WASHINGTON, DC

**Keywords:** American Telemedicine Association, Habilitation, Rehabilitation, Telehealth, Telepractice

## Abstract

Telehealth is a broad term used to describe the use of electronic or digital information and communications technologies to support clinical healthcare, patient and professional health related education, and public health and health administration. Telerehabilitation refers to the delivery of rehabilitation and habilitation services via information and communication technologies (ICT), also commonly referred to as” telehealth” technologies. Telerehabilitation services can include evaluation, assessment, monitoring, prevention, intervention, supervision, education, consultation, and coaching. Telerehabilitation services can be deployed across all patient populations and multiple healthcare settings including clinics, homes, schools, or community-based worksites. This document was adapted from the American Telemedicine Association’s (ATA) “A Blueprint for Telerehabilitation Guidelines” (2010) and reflects the current utilization of telerehabilitation services. It was developed collaboratively by members of the ATA Telerehabilitation Special Interest Group, with input and guidance from other practitioners in the field, strategic stakeholders, and ATA staff. Its purpose is to inform and assist practitioners in providing effective and secure services that are based on client needs, current empirical evidence, and available technologies. Rehabilitation professionals, in conjunction with professional associations and other organizations are encouraged to use this document as a resource for developing discipline-specific standards, guidelines, and practice requirements.

## INTRODUCTION

The ATA Telerehabilitation Special Interest Group (SIG) is comprised of healthcare practitioners and technology specialists who are engaged in applying information and communication technologies (ICT) in practice to improve access to rehabilitation and habilitation services. This document was developed collaboratively by members of the Telerehabilitation SIG, with input and guidance from other practitioners in the field, strategic stakeholders, and ATA staff. This document was adapted from the American Telemedicine Association’s (ATA) “A Blueprint for Telerehabilitation Guidelines” (2010) and reflects the current utilization of telerehabilitation services. Key updates in this document include: (1) provides clarification on scope and definitions; (2) expands upon the administrative principles, including use of an informed consent and consideration of billing and coding within different types of reimbursement models; (3) enhances the clinical principles; and (4) updates the technical principles. The purpose of this guide is to inform and assist stakeholders including but not limited to healthcare agencies, administrators, providers, engineers, and information technology support staff in providing effective, quality services that are based on client needs, current empirical evidence, and available technologies. The content in this document addresses general administrative, clinical, technical and ethical principles and not specific practice guidelines for implementing telerehabilitation services. This guide is not intended to replace the primary practitioner’s clinical decision-making about the appropriate course of healthcare for any client. Inherent to this document is the recognition that following best practice standards and promoting effective rehabilitation and habilitation services delivered through ICT may require professional development and training. Furthermore, the material in this guide should not be interpreted, nor used, as a legal standard of care. The content of this document, while divided into administrative, clinical, technical, and ethical content areas, ideally should be considered in its entirety.

## SCOPE AND DEFINITIONS

Telehealth is a broad term used to describe the use of electronic information and telecommunications technologies to support clinical healthcare, patient and professional health related education, public health and health administration. Terminology used to describe telerehabilitation is similarly broad. Some terms specifically refer to individual rehabilitation disciplines, (e.g., tele-audiology, telespeech, teleoccupational therapy, and tele-physical therapy). More generic terms, such as teletherapy, telehealth (endorsed by the American Occupational Therapy Association and the American Physical Therapy Association), and telepractice (endorsed by the American Speech-Language-Hearing Association) are also used, allowing for a broader focus on populations and activities, such as educational settings and wellness promotion in addition to rehabilitation. It is not the intent of this document to resolve the debate over terminology; rather to provide consistency across applications, regardless of vocabulary. For the purposes of this document, the term ‘telerehabilitation’ will be used. The reader is reminded that terminology may differ according to the application and across practice settings and business models. Telerehabilitation refers to the delivery of rehabilitation and habilitation services via a variety of ICT or commonly referred to as, “telehealth” technologies. Clinically, the term ‘telerehabilitation’ encompasses a range of rehabilitation and habilitation services that include evaluation, assessment, monitoring, prevention, intervention, supervision, education, consultation and coaching. ICT used to deliver rehabilitation and habilitation services may incorporate but are not limited to video and audio conferencing, chat messaging, wearable technologies, sensor technologies, patient portals or platforms, mobile health applications, virtual reality, robotics, and therapeutic gaming technologies. ICT usage in the delivery of rehabilitation services is expected to change as technology continues to evolve. Telerehabilitation services are delivered to adults and children by a broad range of professionals. These professionals may include, but are not limited to, audiologists, nurses, occupational therapists, physical therapists, physicians, psychologists, rehabilitation engineers, speech-language pathologists, and educators. Although other personnel such as paraprofessionals, family members, and caregivers may be involved in telerehabilitation encounters, for the purposes of this document, the term ‘professionals’ will be used to denote rehabilitation professionals using telerehabilitation services. The term ‘clients’ will be used to refer to all recipients of telerehabilitation services and is intended to include both children and adults. The delivery of telerehabilitation services to clients can be implemented across multiple settings that may include, but are not limited to, healthcare settings, clinics, homes, schools, community settings, or community-based worksites.

## KEY PRINCIPLES

The following information represents key administrative, clinical, technical, and ethical principles that should be considered in the deployment or integration of telerehabilitation services. Some principle statements may be repeated in more than one category as relevant. As education and advocacy are central to the continued growth of telehealth, this document serves as a resource to educate members of health professions, students, stakeholders, administrators, legislators, and community members. Rehabilitation professionals, in conjunction with professional associations and other organizations are encouraged to use this document for guidance in developing discipline-specific standards, guidelines, professional development or training, and practice requirements. A bibliography of research related to telerehabilitation can be found on the webpage of the ATA Telerehabilitation SIG (www.americantelemed.org). Additional key telerehabilitation-related guidance documents can be found on the websites of the American Occupational Therapy Association, American Physical Therapy Association, and the American Speech-Language-Hearing Association. Telerehabilitation stakeholders should also refer to the ATA Core Operational Guidelines for information that pertains to general telehealth operations. This resource has been updated to reflect contexts specific to telerehabilitation. This document contains requirements, recommendations, or actions that are identified by text containing the keywords “shall,” “should,” “may” and “shall not.” “Shall” indicates a required action whenever feasible and practical under local conditions. These indications are found in bold throughout the document. “Should” indicates and optimal recommended action that is particularly suitable without mentioning or excluding others. “May” indicates additional points that may be considered to further optimize the healthcare process. “Shall not” indicates that this action is strongly advised against.

### ADMINISTRATIVE PRINCIPLES

- Organizations and professionals **shall** be aware of and comply with laws, regulations, guidelines, and standards set forth by nationally recognized professional associations and other credentialing, privileging, accrediting, and regulatory requirements for licensing, certification, professional liability, and ongoing professional development or training for use of ICT for delivering provisional services and products.
- Organizations and/or professionals **shall** be aware of and comply with any federal or state laws or licensure regulations that contain language that **may** restrict types of services that can be delivered by ICT or promulgate any other specific limitations or requirements of provisionary services when delivered by ICT.
- Organizations and professionals **shall** be aware of all applicable models of licensure portability (e.g., Interstate Licensure Compacts, Expedited License, Limited License, etc.) that may impact interstate practice using ICT.
- Organizations and professionals **shall** be aware of and comply with any additional or necessary operational or contractual requirements or arrangements with the implementation of telerehabilitation services. Examples include contractual agreements (e.g., service level agreements, Business Associate Agreements) and credentialing requirements at the site where the practitioner is located and the site where the client is located (which may vary between states or jurisdictions).
- Organizations and/or professionals **shall** be aware of and comply with or follow any federal and state telehealth laws, professional regulations or organization’s policies in regards to the informed consent. The informed consent informs and educates the client regarding the scope of the telerehabilitation services during a virtual encounter and can include types of services; types of technologies; capturing of video, audio, and/or photos; use of support personnel; record keeping; privacy and security; terminating services; billing arrangements; and other parameters related to encounters.
- Organizations and professionals **shall** be aware of current billing and coding processes (e.g., the use of any designated modifiers or specific codes) that may be required by federal or state insurance plans, commercial insurers, or private health plan payment policies. Providers **shall** be aware of various reimbursement arrangements, agreements and alternative payment methodologies that now exist such as fee for services, bundled care, primary care medical home, accountable care organizations, shared savings, partial risk and other payment models with cost savings and cost avoidance metrics. This could affect the scope of services implemented and/or the billing and coding methodologies.
- Organizations and/or professionals **shall** be aware of and comply with the advanced requirements for privacy, security, and confidentiality (e.g., HIPAA, HITECH, FERPA, etc.) associated with delivering telerehabilitation services. This **may** include orientation to organization’s policies governing the use of ICT, the appropriate use of devices, and privacy and security considerations prior to engaging in telerehabilitation services.
- Organizations and professionals **shall** be aware of and comply with all federal, state, professional association, and healthcare entity requirements for clinical and nonclinical documentation.
- Organizations and professionals **shall** have a mechanism in place to ensure that clients are aware of their rights and responsibilities with respect to accessing telerehabilitation services and/or their personal health records including the process for communicating complaints and grievances.
- Organizations and professionals **shall** ensure that an appropriate facilitator, telepresenter, translator or e-helper (caregiver, family member, provider or another authorized individual) is available when necessary to meet client needs before, during, and after the telerehabilitation encounter. However, any additional personnel or persons accompanying or participating in the virtual encounter **shall** be announced, recognized, and approved by the client and the provider.
- Organizations and professionals **shall** consider establishing mechanisms or policies and procedures to determine the client’s location at the time of the virtual encounter, to implement secondary modes of communication (e.g., telephone or text) in the event of technical or communication disruption during the encounter, and to promote an emergency plan for ensuring the client’s safety in the event of a health-related complication.
- Organizations and professionals engaged in telerehabilitation research **shall** ensure the protection of participants in research protocols. Research protocols **shall** be approved by the research/ethical review committee (e.g., Institutional Review Board) of the affiliated agency and be in compliance with relevant legislation, regulations, and other requirements for supporting participant decision-making and informed consent.
- Organizations and/or professionals **shall** have in place a systematic quality improvement and performance management process that complies with any organizational, regulatory, or accrediting requirements for outcomes management. The organization **should** review the telerehabilitation program on a periodic basis to identify risks, quality of service, and continued viability of the program. Assessment metrics **may** include quality of equipment and connectivity, client and professional satisfaction with virtual encounters, clinical outcomes, appropriateness of the virtual encounter, review of clinical documentation quality (e.g., chart review/audit) and other performance and quality metrics.
- Organizations and professionals that engage in collaborative partnerships and/or vendor agreements **shall** be aware of applicable legal and regulatory requirements for appropriate written agreements such as, but not limited to, Memorandum of Understanding (MOU), Master Service Agreements (MSA) or contracts such as the Business Associate Agreement (BAA). The agreements **shall** be based on the scope and application of the telerehabilitation services offered and **shall** address the administrative, clinical, technical, ethical, and privacy requirements outlined in this document, as relevant, for all parties named.

### CLINICAL PRINCIPLES

- Professionals **shall** be aware of and comply with laws and regulations and **shall** integrate guidelines, and standards set forth by nationally recognized professional associations (e.g., American Speech-Language Hearing Association, American Physical Therapy Association, and American Occupational Therapy Association) and other credentialing, privileging, accrediting, and regulatory requirements for licensing, certification, professional liability, and ongoing professional development or training for use of ICT for delivering provisional services and products.
- Professionals **shall** be aware of and comply with all professional state board regulations and any guiding scope of practice policies.
- Professionals who use ICT hardware, software, or devices to deliver information or services should be trained in equipment and software operation and troubleshooting or readily have available the technology vendor or IT support to assist with technical difficulties. Providers should also know how to operate videoconferencing peripherals, such as data sharing tools, to incorporate and modify education, intervention, materials or accessories used during an encounter.
- Professionals performing telerehabilitation services whether synchronous (real-time interactive) or asynchronous (store-and-forward) **shall** consider the need to modify education or intervention materials, techniques, equipment, and/or the physical setting. Regardless of any modifications made, professionals **shall** deliver services in accordance with professional standards of care, copyright laws, and the principles of evidence-based practice.
- Professionals **shall** be aware of and comply with all federal, state and professional regulations and any additional healthcare entity requirements for clinical and non-clinical documentation, collection and storage of health data, health and/or medical record storage and management, and retrieval or sharing of client data or medical records to protect the client’s personal health information in accordance with federal and state regulations (e.g., HIPAA, HITECH, FERPA).
- Professionals **shall** ensure that an appropriate facilitator, telepresenter, translator or e-helper (caregiver, family member, provider or another authorized individual) is available when necessary to meet client and provider needs before, during, and after the telerehabilitation encounter. However, any additional personnel or persons accompanying or participating in the virtual encounter **shall** be announced, recognized, and approved by the client and the provider.
- Professionals assume responsibility for ensuring the client’s safety during telerehabilitation service encounters. If during the virtual encounter, the professional observes the client might be experiencing any medical symptoms, complications, or emergency, the virtual encounter **shall** be terminated and the client referred to an appropriate local healthcare provider or emergency services according to established policy and procedure.
- Professionals **shall** be aware of and comply with any applicable organizational or administrative telehealth guidelines impacting the delivery of telerehabilitation services.

### TECHNICAL PRINCIPLES

- Organizations and professionals **shall** comply with all relevant laws, regulations, and codes for technology and technical safety.
- Organizations and professionals **shall** ensure that equipment is safe and sufficient to support diagnostic and/or treatment needs and is functioning properly at the time of clinical encounters. This includes having available additional types of technologies or peripheral devices (e.g., measurement tools, sound meters, sensor technologies, etc.) that **may** be necessary to provide evaluations and interventions.
- Organizations and professionals **shall** have infection control policies and procedures in place for the use of telehealth equipment and peripherals that comply with organizational, and/or national, state and local regulatory requirements. In particular, mechanisms **shall** be in place for the cleaning/sterilization of equipment for re-use by multiple clients.
- Organizations and professionals **shall** comply with federal and state regulations (e.g., HIPAA, HITECH, FERPA) for protection of client health information and to ensure the usage of privacy and security measures for protection of data and record storage, retrieval, and transmission and to ensure physical security of ICT hardware, software, and devices. Methods for protection of privacy and security of health information **may** include the use of authentication and/or encryption technology, audit trails, and role based access control.
- Organizations and professionals **shall** have policies and strategies in place to address any modifications to the physical environment, hardware, software applications, and/or peripheral devices and should consider usability and accessibility factors of the client (e.g., fine/gross motor skill, behavioral aspects, cognition, speech, language, vision, and/or hearing) to promote quality services and outcomes.

### ETHICAL PRINCIPLES

- Organizations and professionals **shall** incorporate organizational values and ethics into policy and procedures related to telerehabilitation.
- Organizations and professionals **shall** be aware of and comply with any applicable laws, regulations, statutes, and/or telerehabilitation-related policies and adhere to professional codes of ethics.
- Organizations and/or professionals **shall** inform clients of their rights and responsibilities when receiving rehabilitation and habilitation services through telerehabilitation, including their right to refuse or discontinue services.
- Organizations and professionals **should** have in place a formal process for resolving ethical issues as well as policies that identify, eliminate, and reduce conflict of interest associated with the provision of telerehabilitation services.

